# Detection of gender-based violence in primary care in Central Catalonia: a descriptive cross-sectional study

**DOI:** 10.1186/s12913-023-09091-6

**Published:** 2023-02-02

**Authors:** Ester Muñoz-Sellés, Georgina Pujolar-Díaz, Aïna Fuster-Casanovas, Queralt Miró Catalina

**Affiliations:** 1 Midwife ASSIR Osona. Institut Català de la Salut, Manlleu, Spain; 2 Research Support Unit of Central Catalonia, University Institute for Primary Health Care Research Foundation Jordi Gol i Gurina, Sant Fruitós de Bages, Spain; 3 Health Promotion in Rural Areas Research Group, Gerencia Territorial de la Catalunya Central, Institut Català de la Salut, Sant Fruitós de Bages, Spain

**Keywords:** Prevalence, Gender-based violence, Primary care, Pregnancy, Mental health

## Abstract

**Background:**

Violence against women is a serious public health problem. Primary care could be one of the ideal places for the detection of gender-based violence (GBV), since women come into contact with PC at some point in their lives to look after their sexual and reproductive health. The increase in initiatives promoted by the health authorities regarding GBV offers the possibility of observing its evolution over the last few years.

**Methods:**

A descriptive cross-sectional study of reported cases of GBV in the region of Central Catalonia, during the period from 2017 to 2021, was carried out. All women of legal age, belonging to the specified health region and suffering episodes of GBV, were included. The variables analysed were age, area of residence, health diagnoses related to GBV, whether or not they were pregnant at the time of the attack, and mental health history.

**Results:**

Of the total number of women studied, 1,467 presented some type of diagnosis of GBV, with a total of 3,452 episodes reported. We found an increase in the detection of cases, although it must be noted that there is an underreporting of cases in PC. The prevalence according to the total number of women assigned per year over the period studied was 0.42% in 2017 and 0.48% in 2021. It has also been observed that the average number of episodes per woman increased from 1.03 in 2017 to 1.15 in 2021. During the 5 years analysed, the minimum number of episodes per woman was 1 and the maximum was 10. In reference to the duration of the episodes, the minimum was 1 day, and the maximum was 32 years. The mean age of the women was 42.10 years, the most frequent nationality was Spanish (46.60%), and 54.15% of them lived in rural areas.

**Conclusions:**

Despite the established protocols and procedures, it seems that primary health care is not the most frequent place for its detection. It is necessary to continue working to raise awareness and train professionals, and to ensure coordination among all the parties involved in accompanying women in these processes.

**Trial registration:**

CEIm: 21/278-P.

## Background

From a global perspective, violence can be defined as an unnecessary, harmful or destructive action or omission by one person towards another that is likely to harm his or her dignity, integrity or freedom. The main component of this form of violence is abuse or aggression, i.e., an action or intention that causes harm to others. Based on this definition, various forms, natures and spheres of violence can be distinguished, although the core of this study will be gender-based violence (GBV), that is, any type of violence exercised against women, simply because they are women. Violence can have physical, psychological, emotional, economic and social consequences, affecting the health of women, their children and their environment, and can even lead to death [1–3].

Furthermore, it is a reality that, among couples or families, intimate partner violence (IPV) is one of its most common forms [4, 5]. This kind of violence occurs within the framework of an affective relationship, which makes it more difficult to identify and get out of it [2, 5]. Types of violence are often mixed simultaneously, as they are not mutually exclusive [1].

In a global context, violence against women is a public health problem and its most prevalent form is found in the intimate partner setting. According to the World Health Organization (WHO), one in three women experience physical or sexual violence in their lifetime, mainly by an intimate partner, or sexual assaults perpetrated by others [6]. In addition, three out of five women die as a result of intimate partner violence; 18 million girls between the ages of 15 and 19 have experienced sexual violence at some point in their lives; 200 million women and girls have been subjected to female genital mutilation; and 650 million women and girls were married before the age of 18 [7].

At the Spanish level, according to the National Institute of Statistics, the number of female victims of GBV in 2021 was 30,141, 3.2% higher than the previous year, placing the largest increase among children under 18 (28.6%) [8]. In 2019, it was estimated that 14.2% of women residing in Spain had suffered physical or sexual violence and 31.9% psychological violence in the intimate partner setting (current or past). 21.7% of them had reported the situation, while only 6.78% had gone to health services for accompaniment and support [9]. At the regional level, in Catalonia, 16,099 reports were filed for GBV in 2019, where 82% were for intimate partner violence and 18% for family violence. Likewise, of the 10 femicides that occurred in 2020, 8 were in the intimate partner setting and 2 in the family setting [10].

Gender-based violence, particularly in the intimate partner setting, is a reality and a concern for healthcare institutions. Women turn to the system, but their demands are often not explicit. They usually go to deal with the after-effects of the abuse, but not the abuse itself, which in most cases remains hidden from the health system for years. Despite the high prevalence of IPV, it is rarely detected and, when it is, it is usually at a late stage [11] and it is clear that the violence being carried out poses a serious risk to the health of women and their children [1, 2].

The impact of the COVID-19 pandemic on GBV should also be taken into account, since it has favoured several mechanisms for its occurrence, especially in situations of confinement and in cases of IPV: isolation of the victim, increased control due to physical confinement and impunity in the proceedings, since mobility is limited. These conditions have made it difficult for the women affected to leave situations of violence due to lack of opportunities and limited access to care resources [12–14].

It is also worth mentioning the case of women who suffer GBV during the gestational period, which is generally perpetrated by their intimate partners. In developed countries, most studies document that prevalence ranges from 3.4 to 8.3% [15–17]. Thus, this type of violence occurs heterogeneously among continents, also showing that it starts or increases during gestation [18], in approximately 60.6% of cases [19]. A study conducted in fifteen public hospitals in southern Spain revealed that, although there are different screening instruments for violence during pregnancy, it is important to assess it in different cultural contexts [20].

Similarly, suffering GBV can have a great impact in terms of mental health. There are several publications that estimate that between 20–48% of women seen in mental health services, primary care (PC) or hospital emergency departments suffer gender-based violence [21–23]. According to the WHO, the most frequent associated clinical diagnoses are depression and/or anxiety, sleep disorders due to post-traumatic stress, eating disorders, suicide attempts and substance abuse, among others, whose consequences may extend to social isolation, loss of employment due to absenteeism from work, etc. [24].

PC could be one of the ideal places for the detection of gender-based violence, since women come into contact with PC at some point in their lives to look after their sexual and reproductive health [25–27]. Women go to PC to be treated for the consequences of violence. In this context, the management by the healthcare professionals is vital in these situations. Creating safe environment is essential for these women to have the opportunity to open up and talk about these sensible situations. Professionals must have the training to detect any signs of violence to evaluate and act according to the individual women’s needs [25]. Therefore, it is important to raise awareness and train professionals in order to improve detection and make adequate records, promoting communication between networks to assess possible situations of intimate partner violence and evaluate them from all possible perspectives [1, 2, 4, 28].

The increase in initiatives promoted by the health authorities regarding gender-based violence offers the possibility of observing its evolution over the last few years. Coinciding with the latest legislation in this area [2], public administrations are placing emphasis on increasing their commitment to cases of violence, emphasising a greater need for awareness and training of health professionals, education, law enforcement and communication professionals, among many others [2]. Protocols are being updated and models of care are being created to review the patriarchal structures inherent in the health care system [29].

For all these reasons, this study is proposed with the hypothesis that the growing awareness and the initiatives promoted in terms of gender-based violence in Catalonia will show an increase in the reporting of cases in gender-based violence and more specifically in IPV (due to the high prevalence). To analyse it, this study aims to describe the situation and the evolution of the prevalence of reported cases of gender-based violence in the Central Catalonia health region during the period 2017–2021. It also seeks to describe the sociodemographic profile of women who have suffered gender-based violence, to determine whether there is a higher incidence in urban or rural areas, as well as the cases and episodes, including pregnancies. Lastly, the study aims to relate episodes of violence to mental health diagnoses.

## Methods

### Design, scope and period of study

Descriptive cross-sectional study of gender-based violence cases reported in the Central Catalonia region, during the period from 2017 to 2021.

Catalonia is one of the 17 autonomous communities that make up Spain, which have legislative powers in health matters. In turn, Catalonia is divided into seven health regions, with the Central Catalonia region being the subject of this study.

### Participants and sample

Cases were considered eligible following the definition of the United Nations, which refers to “any act of gender‐based violence that results in, or is likely to result in, physical, sexual or psychological harm or suffering to women, including threats of such acts, coercion or arbitrary deprivations of liberty, whether occurring in public or private life” [3, 30]. The variables considered as cases of GBV were codified according to the World Health Organization’s International Statistical Classification of Diseases and Related Health Problems, Tenth Revision (ICD-10) [31].

All women of legal age, belonging to the specified health region, with episodes of GBV diagnosed in the E-CAP program (health management program used by Catalan Health Institute (ICS) professionals) during the previously specified period were included (see Table 1). Data were retrieved from Primary Health Care Teams and Sexual and Reproductive Health Care Services (ASSIR). A specific database was created with the information requested from the technical service of the Central Catalonia Management of the ICS. Each patient was assigned a number, to eliminate any non-anonymized information about them, and their demographic data, associated diagnoses of violence and recurrent episodes, as well as diagnoses and episodes of mental health, were collected.

**Table 1:** List of gender-based violence codes

C01-Z63.0	Relationship problems with the spouse or partner
C01-O9A.31	Physical abuse complicating pregnancy
C01-T74.01	Adult neglect or abandonment
C01-T74.11	Adult physical abuse
C01-T74.21	Adult sexual abuse
C01-T74.31	Adult psychological abuse, confirmed
C01-T76.01	Neglect or abandonment of the adult, suspicion
C01-T76.11	Adult physical abuse, suspicion
C01-T76.21	Adult sexual abuse, suspicion
C01-T76.31	Adult psychological abuse, suspicion
C01-Y07.01	Husband, perpetrator of abuse and neglect
C01-Y07.03	Male partner, perpetrator of abuse and neglect
C01-Z04.71	Assistance for examination and observation following suspected physical abuse of adults
C01-Z63.0	Relationship problems with the spouse or partner
Y07.0	Partner abuse syndrome
Z63.0	Problems in the relationship between spouses

Obtained from International Statistical Classification of Diseases and Related Health Problems, Tenth Revision (ICD-10), World Health Organization [31]

The total number of women of legal age registered in that health region was also requested in order to perform a prevalence-based data analysis. The average annual number of women registered in the study area was 170,617 women. Exclusion criteria were male and female minors, as well as health problems not caused by gender-based violence.

### Variables

The variables analysed were age, area of residence in order to group women in urban or rural areas according to population of more than 10,000 inhabitants [32], health diagnoses related to GBV (Table 1), whether or not they were pregnant at the time of the attack, and their history of mental health problems.

### Data analysis

For the calculation of prevalence, the number of women and diagnosed episodes of both violence and mental health were differentiated. There could be several episodes, and these could have a start and end record date in the same year or remain open at the end of the study. With this, the prevalence was estimated by dividing the number of women by the total number of women assigned.

To describe the variables, absolute frequencies and percentages were used in the case of categorical variables, and mean with deviation or median with quartiles in the case of continuous variables. To analyse the relationship between two variables, the X2, Student's t-test or Mann–Whitney test was used depending on the type of variable studied.

The significance level was set at 5% and all confidence intervals were at 95%. All analyses were performed with version 4.0.3 of the statistical software R.

## Results

The average annual number of women of legal age assigned in the Central Catalonia region was 170,617 women during the five years analysed. Of the total number of women, 1,467 presented some type of diagnosis of gender-based violence in the period studied, with a total of 3,452 episodes reported. Some of the episodes were closed during that period, while others remained open until after 2021.

**Fig. 1:**
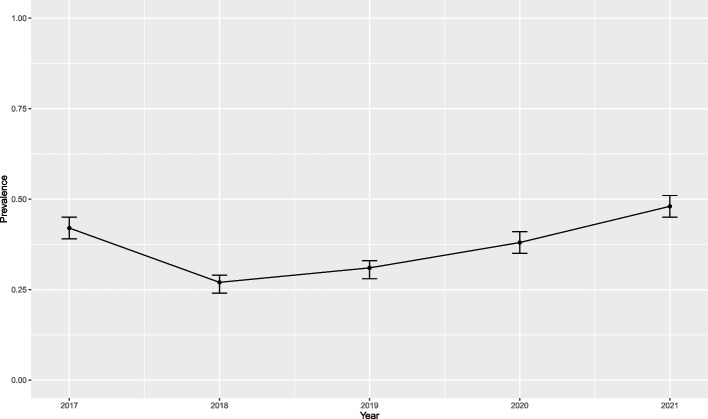
Annual prevalence of diagnoses associated with gender-based violence

Table 2 shows that the prevalence according to the total number of women assigned per year over the period studied was 0.42% in 2017 and 0.48% in 2021. In addition, it can be seen in the same table and graphically in Fig. 1 that the prevalence in 2021 was significantly higher than in 2020 and the initial 2017 (*p*-value < 0.001 in both contrasts). Also noted in the same table is the average number of episodes per woman, increasing from 1.03 (1.01; 1.04) in 2017 to 1.15 (1.12; 1.18) in 2021. During the 5 years analysed, the minimum number of episodes per woman was 1 and the maximum was 10 (Max = 10; Min = 1; X = 1.46; SD = 0.79). In reference to the duration of the episodes, the minimum was 1 day, and the maximum was 32 years (Max = 32 years; Min = 1.5; X = 3.3; SD = 4.05).

**Table 2:** Characteristics of gender-based violence in Central Catalonia, 2017–2021

	**Women assigned**	**Women with a minimal episode**	**Prevalence**	**95% CI of prevalence**	**Number of episodes**	**Average number of episodes per woman—year**	**95% CI of the mean**
2017	167,386	704	0.42%	(0.39; 0.45)	723	1.03 (0.17)	(1.01; 1.04)
2018	168,893	452	0.27%	(0.24; 0.29)	468	1.03 (0.18)	(1.02; 1.05)
2019	171,218	532	0.31%	(0.28; 0.33)	562	1.06 (0.26)	(1.03; 1.08)
2020	171,930	654	0.38%	(0.35; 0.41)	735	1.12 (0.41)	(1.09; 1.15)
2021	173,659	837	0.48%	(0.45; 0.51)	961	1.15 (0.45)	(1.12; 1.18)

The mean age of the women with a diagnosis of GBV was 42.10 years (SD = 15.90; min = 18; max = 97), with the highest number of diagnoses in the 50–60 age group. The most frequent nationality was Spanish (46.60%), followed by Moroccan (9.20%) and, grouped together, several South American nationalities (9.90%). 29.70% are reported as unknown nationality. Furthermore, 54.15% of the women diagnosed lived in rural areas (Table 3).

**Table 3:** Sociodemographic variables of women with a diagnosis of gender-based violence

Variable	N (%)
Age (mean, SD)	42.10 (15.90)
Nationality	
Spanish	684 (46.60%)
Moroccan	135 (9.20%)
South American*	130 (9.90%)
Unknown	435 (29.70%)
Others	83 (5.60%)
Rurality	
Rural area	795 (54.15%)
Urban area	672 (45.85%)

Of the diagnoses analysed, the most relevant are described in Table 4, where it can be seen that 51% referred to relationship problems with the partner, 20.49% to confirmed physical abuse, 12.67% to partner abuse syndrome, 3.51% to sexual abuse, 2.15% to confirmed psychological abuse, 2.12% to suspected psychological abuse and 1.16% to suspected physical abuse, among others.

**Table 4:** Description of the diagnoses analysed

Diagnosis	N (%)
Relationship problems with a spouse or partner	1759 (51.00%)
Spousal or partner abuse syndromes	437 (12.67%)
Adult physical abuse, confirmed, initial assistance	365 (10.58%)
Unspecified adult abuse, confirmed, initial assistance	204 (5.91%)
Adult abuse, confirmed, initial assistance	121 (3.51%)
Adult physical abuse, confirmed	77 (2.23%)
Adult psychological abuse, confirmed	74 (2.15%)
Adult psychological abuse, suspicion	73 (2.12%)
Unspecified adult abuse, confirmed	61 (1.77%)
Adult physical abuse, suspicion	40 (1.16%)
Others	238 (6.90%)

Of the total number of women with a diagnosis, 151 pregnancies were recorded during the period analysed. Of these, 73.51% of women (*n* = 111) had some episode of violence while pregnant, of which 3 had a record of pregnancy complication. Of all pregnancies, 7.95% (*n* = 12) ended with a first trimester abortion and 1.32% with a second trimester abortion. 7.95% of the pregnancies resulted in preterm delivery (*n* = 12), 78.15% (118) in term delivery and 4.64% were unclosed episodes, the development of which is unknown.

Regarding mental health (MH), 58.69% (*n* = 861) of women with diagnoses of violence had some type of related MH diagnosis. In total, 1,251 episodes of reported MH diagnoses were identified. The most prevalent were 56.94% anxiety disorder (*n* = 713); 33.49% major depressive disorder (*n* = 419); 6.07% of cases with injuries (*n* = 76); 2.95% suicide attempts (*n* = 37); and 0.4% others (*n* = 6). The minimum duration of active MH diagnoses was 58 days (Mx = 42.13 years; Md = 6.26; X = 7.25; SD = 5.68).

## Discussion

This study has made it possible to describe the situation and the evolution of the prevalence of reported cases of GBV in the Central Catalonia health region during the period 2017–2021. During the five years of analysis of this study, a total of 1,467 women were treated for violence in primary health care, in an average annual population of 170,617 women.

The prevalences obtained per year range from 0.27% to 0.48%, with an increase in both reported cases and average number of episodes per woman/year from 2018 onwards. The prevalence of women who have experienced physical and/or sexual violence (both within and outside the intimate partner setting) is as high as 22% in Europe and up to 30% worldwide, much higher than the data revealed in this analysis [33]. Also in relation to episodes, in a published study on the effectiveness of psychological treatment for abusers, the mean number of episodes in the last year of cohabitation with the partner was between 1.75 and 23,37 episodes of minor physical aggression according to aggressor profiles between low and high risk [34], a fact that is found in our study, where several women were found with episodes of many years' duration, one of a maximum of 32 active years. Furthermore, taking into account that the impact of the COVID-19 pandemic has been assessed, since it has favoured several mechanisms for the occurrence of violence [12–14]. Despite observing a lower number of cases reported considering the current literature on this topic [33], the results obtained seem consistent with this situation, showing an increasing trend of cases of violence during the years 2020 and 2021 [12].

In the context of the Catalan health system, there is an action protocol in the health services for a coordinated intervention in the prevention, detection, care and recovery of these cases [35]. Current legislation establishes the importance of raising awareness and training healthcare personnel in order to improve detection and record keeping [1, 2, 5]. PC is the access door for women suffering from GBV, usually to treat the consequences of violence instead of the violence itself. Thus, it is crucial to detect signs of violence proactively, in order to provide early and better care. Likewise, the health system must guarantee and adequate work environment for professionals, with appropriate structures and resources to accompany women and children during these processes [25].

However, the results obtained in Central Catalonia might suggest that primary care is not the most usual setting for its detection [36]. Several factors may influence this lack of identification, as well as the prolonged duration of episodes. At the practitioner level, poor training can lead to a less proactive attitude to research, as well as limited time available for consultation and limited personal tools. For women, the normalisation of violence, tolerance of repetitive cases, lack of trust in the system and/or communication difficulties may play a role. From the point of view of the system, it could be associated with a lack of resources, changes in episode reporting codes and inadequate coordination between the different parties involved [1, 35].

Currently, most cases of GBV are detected by the Social Services, the Specialized Intervention Services (SIE) or the Information and Assistance Service for Women (SIAD) of the different municipalities, which are not part of the health system. Although the procedure planned by the government of Catalonia provides for coordination between the different sectors involved, the data collected in PC and the aforementioned services differ significantly. In 2020, Central Catalonia PC centres detected 654 cases, while the various Social Services teams detected 1,112 cases. The SIEs treated 1065 women and the SIADs detected 976 cases [37]. Therefore, there are possibly some difficulties in the procedure planned by the Catalan institutions to coordinate the different referents, women’s care centres and case follow-up.

Regarding the profile of the victims, the literature on IPV has described that women living in rural areas, immigrants or elderly women are some of the most susceptible profiles to experience this type of violence [1]. However, several authors point to the unpredictability of violence, where all women can be victims of it, and such violence can occur in any social sector [38–40]. In this sense, this analysis shows certain heterogeneity in the results obtained according to primary care data in Central Catalonia, coinciding with the latter perspective: the mean age is 42 years (SD = 15.9), although the 50–60 age range is where more cases are detected; 54.15% of cases lived in rural areas and 19.1% were migrants (mainly from Morocco and South America). These results also coincide with a survey conducted in 2016 by the Generalitat de Catalunya, where it was observed that the profile of women had an average age of 44.7 years and 10.85% of them were migrants (mostly from South America and North Africa) [41].

Considering that IPV in itself is a serious public health problem, during pregnancy it affects not only the mother but also the foetus. Most research has found that between 3 and 9% of women experience some form of violence during pregnancy, mostly psychological, with rates as high as 50% depending on risk factors such as age, race and poverty level [42, 43]. In this study, a high prevalence of episodes of violence during pregnancy (73.51%) was found among women who had already suffered a previous episode. This type of violence can cause several complications, such as foetal growth restriction, premature delivery, haemorrhage during pregnancy and perinatal death [19]. The data obtained in this analysis show 7.95% first trimester abortions and 1.32% second trimester abortions, as well as 7.95% preterm deliveries. It is noteworthy that 4.64% of the episodes, although this is a small prevalence, corresponds to non-closed episodes, i.e., the health services do not know how the case evolved, which indicates a lack of communication between the possible parties that could have intervened in the case.

Another aspect where GBV has a great impact is on the mental health of the women who suffer it. Several studies have analysed the relationship between suffering this type of violence and suffering or developing mental health disorders. Likewise, some publications estimate that between 20–48% of women seen in mental health, primary care (PC) or hospital emergency departments suffer GBV [22, 23]. The results of this study show that 58.69% of the cases presented some type of mental health-related diagnosis, either before or after the episode of violence. The most prevalent disorder was anxiety (56.94%), followed by major depressive disorder (33.49%), with lower prevalence in cases of injury or suicide attempts. These results are consistent with WHO estimates, which reveal that the most frequent associated clinical diagnoses are depression and/or anxiety, post-traumatic stress sleep disorders, eating disorders, suicide attempts and substance abuse, among others, whose consequences may extend to social isolation, loss of employment due to absenteeism, etc. [24]. However, due to the persistent stigma surrounding mental health, it can be difficult to detect mental health disorders as a consequence of GBV. Similarly, having multiple pathologies can revictimize some women, especially in those environments where there is insufficient training and awareness to support this type of patient [44].

Considering that women come into contact with PC at some point in their lives, this should be a detection site with a significant role [11, 23]. It is crucial that the sectors involved and areas related to violence are in constant training, and that the information channels are communicated, updated and streamlined, in order to be able to address cases from a multidisciplinary perspective, since the impact on the lives of people who suffer it affects multiple areas (health, education, law enforcement, social services, etc.) [2]. Therefore, with the findings of this study, it can be considered that current protocols and legislation still fail to guarantee this joint approach, and communication between the services involved in prevention, detection, follow-up and reparation should be improved to guarantee the type of support that the affected women need. In addition to clear guidelines and procedures, there is a need for more training and awareness in society in general, and to ensure that people are educated on equality from an early age. The social approach to GBV must be changed at a younger stage of life, where it is crucial to educate on the prevention of sexist attitudes from school in order to avoid focusing all the attention only on the victims [26].

### Strengths and limitations of the study

There are some local studies on the prevalence of violence, but this is the first such study in the region. During these years, the list of diagnostic codes for reporting violence has been extended, so that professionals have more chance to register cases, and there are more possibilities to objectify it. In recent years, at the level of Catalonia, resources have been allocated to the development of strategies and protocols, training of professionals and coordination of teams, but there is still a limitation of reported versus actual cases. On the one hand, the results suggest that women suffering violence do not go to primary care centres in these situations. On the other hand, many professionals are not yet aware of the issue and do not take active measures in prevention, detection and follow-up. We noted an underreporting of cases. This can be explained because of a limitation of the health system itself: a shortage of health professionals, with overcrowded consultations, and little time to attend to users. This situation has a direct impact on the care offered, especially when professionals are not enough aware of the seriousness of the issue and the GBV cases might go unnoticed. Thus, a proactive attitude is essential, since violence is not an isolated and one-off event.

There are still limitations in the existence of specialised teams and coordination of the different parties involved in situations of violence. There is also no interconnection between the public and private health care systems.

## Conclusions

This study shows that the prevalence of reported cases of gender-based violence in the Central Catalonia health region increased during the period 2017–2021. However, the data are lower in comparison with other institutions that respond to GBV in Catalonia, which suggests a lack of coordination between the different areas where it is detected. Despite the existence of protocols and health care models, the procedures for detection and treatment of GBV must be improved among all the parties involved, and communication between them must also be improved in order to address cases in a coordinated and accurate manner. It is also necessary for primary care to have more training for professionals and more awareness, and to be more proactive in terms of actively seeking out situations of violence. This would allow them to provide adequate follow-up and continuous support and not only on one-off occasions, since the repercussions are multiple, serious and affect the whole of society.

## Data Availability

The data sets generated and/or analysed during the current study are not publicly available due to women's confidentiality but are available from the corresponding author upon reasonable request.

## Abbreviations

ASSIR: Sexual and Reproductive Health Care Services
E-CAP program: Health management program used by professionals from ICS
GBV: Gender-based violence
ICS: Catalan Health Institute
IPV: Intimate partner violence (violence occurring within both a partner and family setting)
PC: Primary Care
SIAD: Information and Assistance Service for Women
SIE: Specialized Intervention Services
WHO: World Health Organization
